# Multiclass Analysis for the Determination of Pharmaceuticals and Their Main Metabolites in Leafy and Root Vegetables

**DOI:** 10.3390/molecules29153471

**Published:** 2024-07-24

**Authors:** Carmen Mejías, Marina Arenas, Julia Martín, Juan Luis Santos, Irene Aparicio, Esteban Alonso

**Affiliations:** Departamento de Química Analítica, Escuela Politécnica Superior, Universidad de Sevilla, C/Virgen de África 7, E-41011 Seville, Spain; cmpadilla@us.es (C.M.); mamolina@us.es (M.A.); jlsantos@us.es (J.L.S.); iaparicio@us.es (I.A.); ealonso@us.es (E.A.)

**Keywords:** pharmaceuticals, metabolites, vegetables, LC-MS/MS, carrot, lettuce

## Abstract

The irrigation of soils with reclaimed contaminated wastewater or its amendment with sewage sludge contributes to the uptake of pharmaceuticals by vegetables growing in the soil. A multiresidue method has been devised to determine five pharmaceuticals and nine of their main metabolites in leafy and root vegetables. The method employs ultrasound-assisted extraction, clean-up via dispersive solid-phase extraction, and analysis through liquid chromatography–tandem mass spectrometry. Box–Behnken design was used to refine variables such as extraction solvent volume, time of extraction, number of extraction cycles, and the type and amount of d-SPE sorbent. The method achieved linearity (R^2^) greater than 0.994, precision (relative standard deviation) under 16% for most compounds, and detection limits ranging from 0.007 to 2.25 ng g^−1^ dry weight. This method was applied to a leafy vegetable (lettuce) and to a root vegetable (carrot) sourced from a local market. Parent compounds were detected at higher concentrations than their metabolites, with the exception of carbamazepine-10,11-epoxide.

## 1. Introduction

Vegetables that grow in agricultural soils irrigated with contaminated reclaimed wastewater or amended with contaminated sludge or compost from wastewater treatment plants can uptake contaminants through their roots [1,2,3]. Reclaimed wastewater irrigation is widely used nowadays and applying sludge to soils offers agronomic advantages due to its content in organic matter [4]. However, unfortunately, wastewater treatments plants are not designed to eliminate emerging contaminants [5]. Consequently, these pollutants can accumulate in crop soils [6]. Once in the soil, pollutants can be taken up by plants, accumulate in roots, or translocate within the plant [7,8,9,10,11]. Accumulating contaminants in leaves and roots can negatively affect crops and could pose potential human health risks, especially when affecting edible plants [12,13]. The assessment of plant uptake requires accurate and sensitive analytical methodologies [14]. In recent years, these methods focused on detecting pesticides in vegetables [15]. Pharmaceuticals’ active compounds are one of the most studied emerging pollutants. However, analytical methods for detecting pharmaceuticals metabolites in vegetables are limited and mainly focus on the parent compound, with less attention to their metabolites. Some of their metabolites can be more toxic, persistent, and concentrated in the aquatic environment than the parent compounds [16]. Various extraction methodologies have been reported for detecting pharmaceuticals in vegetables, including ultrasound-assisted extraction (UAE) [17,18], pressurised solvent extraction (PLE) [19,20], matrix solid-phase dispersion (MSPD) [21], and QuEChERS (quick, easy, cheap, effective, rugged, and safe) method [22,23]. Extract clean-up is mainly performed using solid-phase extraction (SPE) [17,18,20,24] or dispersive solid-phase extraction (d-SPE) [25]. However, sample treatment generally involves solid–liquid extraction and SPE clean-up, followed by liquid chromatography–tandem mass spectrometry (LC–MS/MS) [25] or gas chromatography–mass spectrometry (GC–MS) [26].

The objective of this study was to develop and validate a multiclass analytical methodology for determining a broad range of pharmaceuticals (five parent compounds from four therapeutic groups) and nine of their main metabolites in leafy and root vegetables. The selected parent compounds, which included caffeine (CAF), diclofenac (DIC), and ibuprofen (IBU), were chosen based on their prevalent usage and consumption; their inefficient removal during wastewater treatment processes, leading to their widespread presence in various environmental compartments; and their potential ecotoxicological risks, as observed with compounds like carbamazepine (CBZ) and sulfamethoxazole (SMX). To the best of our knowledge, this is the first method to quantify pharmaceutical metabolites for different therapeutic groups in vegetables.

## 2. Results and Discussion

### 2.1. Method Optimisation

The method was optimised with spiked carrot samples (0.5 g) at 100 ng g^−1^ dry weight (dw). Samples were individually spiked with the compounds using a methanol solution to ensure thorough permeation of the entire sample. The samples were then vortex-mixed for homogenisation and incubated in the dark for 12 h to allow for proper evaporation of the solvent and equilibration. For the optimisation of d-SPE sorbent, the spiked procedure took place after extraction and before the addition of the clean-up sorbent.

#### 2.1.1. Optimisation of the Extraction Solvent

**Figure 1:**
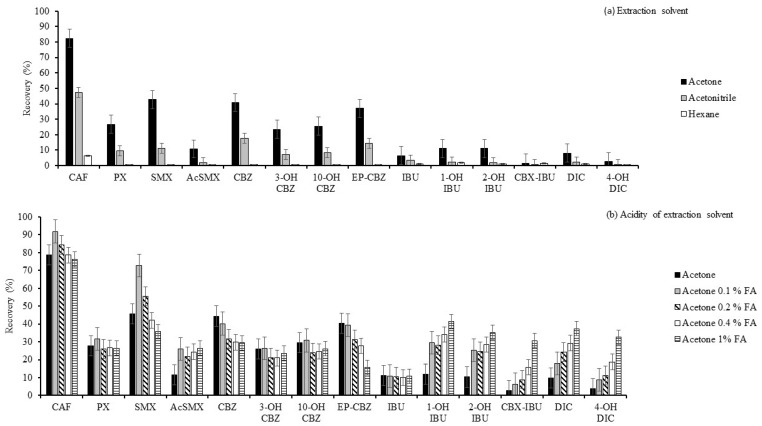
Optimisation of (**a**) extraction solvent and (**b**) acidity of extraction solvent (n = 3).

Extraction solvents tested were acetonitrile (ACN), acetone, and hexane. Methanol (MeOH) was initially included as a tested solvent; however, methanol extracts were characterised by the highest extraction of matrix components, making the handling of the extracts difficult and, thus, they were not analysed further. Samples were extracted three times in an ultrasonic bath for 10 min, centrifuged at 2900× *g* for 10 min, and subjected to clean-up by d-SPE with 0.8 g of C18. Optimisation was carried out in triplicate with 3 mL of the tested solvent. Extraction recovery was calculated by comparison of the signal of the spiked vegetable with a matrix-matched standard at the same spiking concentration. As can be seen in Figure 1a, the best extraction recoveries were obtained with acetone, so acetone was preselected.

Then, different percentages of acidity of acetone were tested (acetone, acetone (0.1% *v*/*v*, formic acid), acetone (0.2% *v*/*v*, formic acid), acetone (0.4% *v*/*v*, formic acid), and acetone (1% *v*/*v*, formic acid)). The extraction procedure was the same as in the optimisation of the solvent. Extraction recoveries were also obtained in the same way as in the previous experiment. As can be seen in Figure 1b, the best extraction recoveries for most compounds, especially for those with lower recoveries, were obtained with acetone (1% *v*/*v*, formic acid), so this solvent was selected as extraction solvent. IBU and metabolites were better extracted at pH < pKa, as they were not ionised at these pH values. The possible evaporation of acetone during the extraction process was evaluated, obtaining no differences in volume.

#### 2.1.2. Optimisation of d-SPE Sorbents and Their Amount

**Figure 2:**
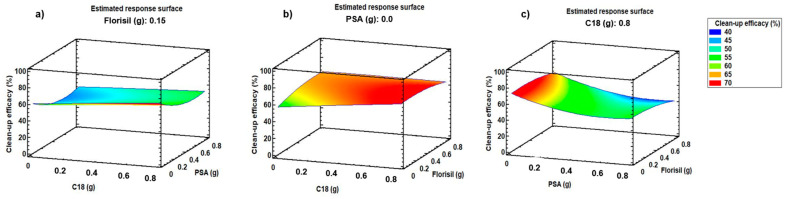
Response surface plots, corresponding to clean-up efficacy (%) of target compounds, versus (**a**) C18 amount (g) and PSA amount (g); (**b**) C18 amount (g) and florisil^®^ amount (g) (**c**); PSA amount (g) and florisil^®^ amount (g).

Clean-up was optimised to select the most appropriate sorbent or sorbents for removal of interfering compounds without removing target compounds. Three clean-up sorbents were tested: a weak anion exchanger sorbent (primary-secondary amine, PSA), a reverse phase sorbent (C18), and a normal phase sorbent (florisil). PSA sorbent is used to remove polar pigments, fatty acids, sugar, and organic acids; C18 is commonly used to remove nonpolar and moderately polar compounds, such as lipophilic compounds; florisil is suitable to remove polar compounds. A Box–Behnken design (BBD) was applied to optimise d-SPE sorbent amount. The variables (types of sorbents: C18, PSA and florisil) were evaluated at three levels (mass of sorbent: 0, 0.4, and 0.8 g). The number of experiments (N) required is defined by the equation: N = 2k (k − 1) + C_0_, where k is the number of variables and C_0_ is the number of central points. In this case, k and C_0_ values were set at 3, resulting in a total of 15 experiments. Samples were extracted three times in an ultrasonic bath for 10 min with 3 mL of acetone (1% *v*/*v*, formic acid) and centrifuged at 2900× *g* for 10 min. Liquid phases of vegetable matrix extractions were spiked at 200 ng mL^−1^ and subjected to the 15 different clean-up experiments. The experiments were randomly performed to minimize the effects of uncontrolled variables. The BBD matrix is shown in Supplementary Materials Table S1. BBD values were calculated using Statgraphics 18-X64 for Windows, using the optimisation of multiple responses mode for the analysis of the results. Clean-up efficacy was evaluated to select the most appropriated sorbent. Higher efficacy is achieved when the tested sorbent removes interferences to a greater extent (inducing less matrix effect) while it does not remove target compounds. Clean-up efficacy was calculated by comparison of the signal of a matrix-matched standard with a standard in pure solvent at the same spiking concentration. Figure 2 shows response surface plots corresponding to clean-up efficacy (%) vs. C18 amount (g) and PSA amount (g) (Figure 2a), clean-up efficacy (%) vs. C18 amount (g) and florisil amount (g) (Figure 2b), and clean-up efficacy (%) vs. PSA amount (g) and florisil amount (g) (Figure 2c). The best values of clean-up efficacy (%) were obtained for 0 g of PSA, 0.8 g of C18, and 0.15 g of florisil. According to the results, 0.8 g of C18 and 0.15 g of florisil were selected as d-SPE sorbents amount for clean-up. Due to its chemical structure, the C18 sorbent was able to remove nonpolar interferences from the extract without removing the selected compounds, making it a suitable sorbent for clean-up.

#### 2.1.3. Optimisation of Extraction Solvent Volume, UAE Time, and Number of Extraction Cycles

**Figure 3:**
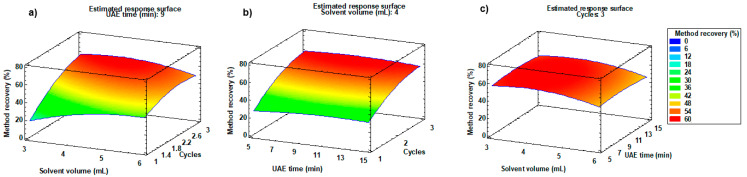
Response surface plots, corresponding to method of recovery (%) of target compounds, versus (**a**) acetone (0.1% *v*/*v*, formic acid) volume (mL) and number of extraction cycles; (**b**) UAE time (min) and number of extraction cycles; and (**c**) acetone (0.1% *v*/*v*, formic acid) volume (mL) and UAE time (min).

As in the optimisation of d-SPE, a BBD was applied to optimise extraction solvent volume, UAE time, and number of extraction cycles. Three levels were evaluated for each variable: acetone (1% *v*/*v*, formic acid) volume (3, 4.5, and 6 mL), UAE time (5, 10, and 15 min), and number of extraction cycles (1, 2, and 3). As in the previous case, 15 experiments were carried out. Details of each experiment can be seen in Table S2 in Supplementary Materials. Samples were subjected to 15 extraction experiments and were cleaned-up with 0.8 g of C18 and 0.15 g of florisil according to the optimisation of d-SPE sorbent and their amount. Response surface plots corresponding to method of recovery were constructed to better evaluate the effects of the extraction method on each variable and their interactions. BBD values were calculated using Statgraphics 18-X64 for Windows, using the optimisation of multiple responses mode for the analysis of the results. Figure 3 shows the response surface plots corresponding to the method of recovery (%) vs. acetone (0.1% *v*/*v*, formic acid) volume and UAE time (Figure 3a), method of recovery (%) vs. acetone (0.1% *v*/*v*, formic acid) volume and number of extraction cycles (Figure 3b), and method of recovery (%) vs. number of extraction cycles and UAE time (Figure 3c). Response surface plots facilitate the optimum value within the evaluated range of each variable, but do not have to coincide exactly with the three values tested in the experiments. As can be seen in Figure 3, number of extraction cycles was the most influential parameter. The best values of method of recovery (%) were obtained for 4 mL of acetone (1% *v*/*v*, formic acid), three extraction cycles, and 9 min of UAE. According to the results, 4 mL of acetone (1% *v*/*v*, formic acid), three extraction cycles, and 9 min of extraction were selected for sample extraction. As can be seen from the results obtained, the number of extraction cycles proved to be the most influential factor in the extraction of the analytes. The extraction time and the volume of solvent ended up having very little influence on the extraction, with it being more of a priority to repeat the process several times than to add a large volume of solvent and to prolong the extraction for a long time.

### 2.2. Method Validation

The method was validated in terms of sensitivity (method detection limits (MDL) and method quantification limits (MQL)), linearity, accuracy (A), and precision. Previously, matrix effect (ME) was evaluated by comparison of calibration curve slopes in pure solvent (external calibration curves) and calibration curve slopes in sample extract (matrix-matched calibration curves). Calibration curves were prepared in triplicate at five concentration levels in the range from 2.25 to 100 ng g^−1^ dw and were constructed using analyte area (axis y) versus analyte concentration (axis x). Student’s *t*-test, at 95% of confidence, revealed statistical differences between slopes so matrix-matched calibration curves were applied for quantification.

The methodology was validated for leafy and root vegetables by using spiked lettuces and carrots. In order to evaluate the linearity, five-point matrix-matched calibration curves were prepared in triplicate in the range from MQL to 100 ng g^−1^. Correlation coefficients (R^2^) were equal or higher than 0.994 for all the compounds in both matrices (Table 1). Instrument detection limit (IDL) and quantification limit (IQL) values were estimated as the concentrations corresponding to signal-to-noise ratios of 3 and 10, respectively, by injecting spiked extracts at low concentration levels. MDL and MQL were calculated from IDL and IQL, applying the concentration factor, matrix effect, and recovery for each compound. MDL values were in the range from 0.002 ng g^−1^ to 1.020 ng g^−1^ dw in lettuces and from 0.007 ng g^−1^ to 0.417 ng g^−1^ dw in carrots. MQL values were from 0.007 ng g^−1^ to 2.25 ng g^−1^ dw in lettuces and from 0.04 ng g^−1^ to 2.25 ng g^−1^ dw in carrots (Table 1).

Recovery and precision were evaluated from spiked samples at three concentration levels (2.5, 40, and 100 ng g^−1^ dw) in triplicate. Recovery (%) was calculated as the percentage of analyte extracted. This percentage was quantified using the signal of a matrix-matched standard as reference to avoid the influence of matrix effect as follows: R (%) = (A_spiked sample_ − A_non-spiked sample_) × 100/(A_spiked extract_ − A_non-spiked sample_). Recovery for most compounds was in the range from 28% to 98% for lettuces and in the range from 26% to 115% for carrots (Table 2). For some analytes, recovery values were higher at the low concentration level; however, the low levels of concentration that were tested (ppt in all cases) could explain this variation. Concerning recovery values, analytical guidelines, like those from the AOAC Peer-Verified Methods program [27], suggest that recoveries can fall between 60% and 120% at part per billion levels, typically referring to “absolute recovery” or “accuracy”. Given that the method proposed in this work is a multi-analyte approach, where compounds with highly varied properties are analysed, it is necessary to achieve a balance that ensures optimal recovery for all analytes. Furthermore, as shown in Table 3, the accuracy reached values between 81% and 107% for all analytes. This parameter reflects the suitability of the method. Precision, expressed as relative standard deviation (% RSD), was determined from the analysis of spiked samples in triplicate on two different days. RSD values were below 16% for most compounds in both types of matrices at the three spiked concentration levels for all compounds. As expected, precision was lower at lower spiking concentrations, because the lower the concentration, the greater the dispersion obtained in the extraction data.

Matrix effect was calculated as follows: ME (%) = (A_spiked extract_ − A_non-spiked extract_ − A_standard_) × 100/A_standard_. Matrix effect ranged from −0.01% to −39.0% in leafy vegetables and from −0.08% to −79.3% in root vegetables (Table 3). In all cases, matrix ion suppression occurred.

Accuracy was obtained using the following equation: A (%) = (C_spiked sample_ − C_non-spiked sample_) × 100/C_spike concentration_. Accuracy percentage ranged from 81% to 107% (Table 3).

In Figure S1 in Supplementary Materials, a chromatogram of a spiked lettuce (10 ng g^−1^ dw) can be seen.

### 2.3. Method Comparison

As can be seen from Table 4, the therapeutic group most studied in vegetables was antibiotics, as well as in the assessment of plant uptake. Most of the methods used 0.5 g of sample amount, but some of QuEChERS methods [22,23] used amounts up to 10 g. Regarding time required for extraction, most methodologies are comparable in duration to the method developed in this work, since several extraction cycles are often necessary for good performance and techniques such as QuEChERS include different procedures. There are just two methodologies that require a significantly shorter time than the one proposed [17,20]. Comparing the type and amount of solvent used, all methods use higher amounts and/or more toxic solvents than acetone, such as ACN, hexane, or MeOH [28]. There are only three methods that require less than 10 mL of extraction solvent [20,22,23], but at the same time using mixtures of ACN and MeOH, and just one not employing organic solvents [17]. All methodologies carry out some clean-up procedure to remove interfering compounds that may affect the analytical determination. Some of them incorporate this clean-up in the same step as the extraction [19,20,22,23], usually in QuEChERS techniques. In other cases, including the proposed methodology, it is performed as an additional stage [17,18,20,24], which, although it adds an extra step to the method, helps to eliminate interferences and to increase the sensitivity and selectivity of the method. Therefore, in terms of green sample preparation, the proposed method can be considered one of the most sustainable when compared to similar reported methodologies for analysing pharmaceutical residues in vegetables. Only one methodology [19] used GC-MS for the determination of the compounds, while the other ones employed LC-MS/MS. The proposed analytical method reported the lowest MQLs (highest sensitivity) in comparison to reported methods in the literature.

### 2.4. Method Application

The method was applied to the determine target compounds in one type of leafy vegetable (lettuce) and one type of root vegetable (carrot). Three carrot samples and three lettuce samples were purchased from local markets and analysed. Concentrations are shown in Supplementary Materials (Table S3). SMX was the compound with the highest detection frequency and highest concentration (up to 7.8 ng g^−1^ dw in carrots). In contrast, 2-hydroxyibuprofen (2-OH-IBU), carboxyibuprofen (CBX-IBU), and 4-hydroxydiclofenac (4-OH DIC) were not detected in any of the analysed samples. In general, parent compounds were found at higher concentrations than their metabolites, with exception of carbamazepine-10,11-epoxide (EP-CBZ). These results show the applicability of the method. The concentrations levels measured were similar to or lower than those reported by other authors for pharmaceuticals in vegetables. As an example, Calderón-Preciado et al. (2009) [19] reported IBU concentration levels in lettuce vegetables of 28.5 ng g^−1^. To our knowledge, this is the first time that concentration levels have been reported for metabolites in lettuce and carrots.

## 3. Materials and Methods

### 3.1. Chemicals and Reagents

Analytical standards of 1,3,7-trimethylxanthine (CAF), 1,7-dimethylxanthine (paraxanthine) (PX), CBZ, 3-hydroxycarbamazepine (3-OH CBZ), 10,11-dihydro-10-hydroxycarbamazepine (10-OH CBZ), EP-CBZ, DIC, 4-OH DIC, IBU, 1-hydroxyibuprofen (1-OH IBU), 2-OH IBU, CBX-IBU, SMX, and N^4^-acetylsulfamethoxazole (AcSMX) were supplied by Sigma-Aldrich (Steinheim, Germany). Physical–chemical properties of the target compounds can be seen in Table S4 in Supplementary Materials. Caffeine-^13^C_3_ (CAF-^13^C_3_) and ibuprofen-d_3_ (IBU-d_3_) used as internal standards were purchased from Sigma-Aldrich (Steinheim, Germany). Individual stock standard solutions were prepared at 1000 μg mL^−1^ in MeOH and stored in amber glass bottles at −18 °C. Working solutions were prepared by dilution of the individual standard solutions or by mixing the individual standard solutions to have a mixture of the target compounds and a mixture of the internal standards (I.S.) Florisil used as d-SPE sorbent was provided by Sigma-Aldrich (Madrid, Spain). PSA and C18 were provided by Scharlab (Barcelona, Spain). HPLC-grade ACN, hexane, and acetone were supplied by Romil (Barcelona, Spain). LC-MS-grade water and MeOH were purchased from Honeywell (Seelze, Germany). Analytical-grade formic acid (98%) was provided by Panreac (Barcelona, Spain).

### 3.2. Sample Collection and Treatment

Root and leafy vegetables were bought in a local market. Carrots were selected as root vegetables and lettuces were selected as leafy vegetables. Fresh and clean vegetables were cut into small pieces, triturated in a blender, freeze-dried in a Cryodos-50 lyophiliser (Telstar, Terrassa, Spain), sieved (particle size < 100 µm), saved in glass bottles, and maintained at −18 °C until extraction. Treatment was based on UAE as the extraction step and d-SPE as the clean-up step. Pre-treated vegetable samples (0.5 g dw) were weighed in glass centrifuge tubes and spiked with I.S. (CAF-^13^C_3_ and IBU-d_3_) at 125 ng g^−1^ dw. This I.S. concentration was selected to be high enough to be observed correctly in all samples to avoid as much as possible the variation of its intensity between samples, since the higher the concentration, the better signal-to-noise ratio and the precision. Samples were extracted with 4 mL of acetone (1% *v*/*v*, formic acid) by sonication for 9 min in an ultrasonic bath at 25 °C. Afterwards, the tubes were centrifuged at 2900× *g* for 10 min, the liquid phase was transferred to a clean tube, and the extraction procedure was repeated twice again. The liquid phase of the three extractions was combined into a clean centrifuge tube and 0.15 g of florisil and 0.8 g of C18 were added for d-SPE extract clean-up. The tubes were vigorously shaken for 1 min and centrifuged for 15 min at 2900× *g*. The liquid phase was transferred to another clean tube and was evaporated to dryness under a gentle nitrogen stream in the XcelVap^®^ system (Horizon Technology, Reading, UK). The dried extract was reconstituted in 250 µL of water–MeOH solution (1:1, *v*/*v*), filtered through a 0.22 µm cellulose syringe filter, and transferred into an automatic injector vial for injection into the LC-MS/MS system.

### 3.3. Liquid Chromatography–Tandem Mass Spectrometry

Chromatographic determination was carried out on an Agilent 1290 Infinity II liquid chromatographic system (Agilent, Santa Clara, CA, USA) coupled to a 6495-triple quadrupole (QqQ) mass spectrometer (MS) equipped with an electrospray ionisation source (ESI). Chromatographic separation was optimised by Malvar et al. (2019) [16] and was performed on a Kinetex^®^ Polar C18 column (50 mm × 3.0 mm i.d., 2.6 µm particle size) (Phenomenex, Torrance, CA, USA) thermostated at 35 °C and protected by a SecurityGuard™ ULTRA C18 guard column (2 mm × 3.0 mm i.d., 2.6 µm particle size) (Phenomenex, Torrance, CA, USA). The injection volume was 10 µL. The mobile phase consisted of water containing formic acid (0.1%, *v*/*v*) (solvent A) and MeOH containing formic acid (0.1%, *v*/*v*) (solvent B) at a flow rate of 0.6 mL min^−1^. A gradient mode was used to separate and elute analytes from the column. Gradient elution started with a linear increase of solvent B from 2% to 38% in 9.5 min (held for 2.5 min), then to 50% in 3.5 min (held for 2.5 min), and, finally, to 98% in 4 min (held for 1 min). The mobile phase returned to initial conditions by linear decrease of solvent B from 98% to 2% in 2 min and held for 5 min for re-equilibration. Total chromatographic run time was 30 min. The ESI-MS/MS conditions were as follows: capillary voltage, 4000 V; drying gas flow rate, 11 L min^−1^; drying gas temperature, 250 °C; sheath gas flow rate, 12 L min^−1^; sheath gas temperature, 250 °C; and nebulizer pressure; 40 psi. The mass spectrometer was operated in dynamic multiple reaction monitoring mode (dMRM). Instrument control and data acquisition were carried out with MassHunter Workstation software version 10.1 (Agilent, Santa Clara, CA, USA). Optimised LC-MS/MS parameters for each compound are given in Supplementary Materials Table S5.

## 4. Conclusions

A new analytical methodology has been optimised and validated for the determination of five pharmaceuticals and nine of their main metabolites in leafy (lettuce) and root (carrot) vegetables. Sample extraction and clean-up are based on UAE and d-SPE, which are low-cost, affordable, and easy-to-perform techniques. Analytical determination is carried out by LC-MS/MS analysis. MQLs values were in the range of 0.007–2.25 ng g^−1^ dw for leafy vegetables and in the range of 0.040–2.25 ng g^−1^ dw for root vegetables. Precision, expressed as relative standard deviation, was lower than 16% for most the compounds. The method was satisfactorily applied, and some pharmaceuticals were detected in both types of vegetables, whereas others were detected just in leafy or root vegetables. The proposed method can provide a useful tool to (i) assess pharmaceuticals uptake in vegetables through soil contamination, (ii) evaluate their accumulation and translocation in plants, and (iii) study human exposure, estimating daily intake of pharmaceuticals by consumption of vegetables.

## Figures and Tables

**Table 1:** Method detection limits (MDL), method quantification limits (MQL), and matrix-matched calibration curve correlation coefficients (R^2^).

Compound	Lettuce			Carrot		
	MDL (ng g^−1^ dw)	MQL (ng g^−1^ dw)	R^2^	MDL (ng g^−1^ dw)	MQL (ng g^−1^ dw)	R^2^
**CAF**	0.011	0.027	0.998	0.022	0.217	0.995
PX	0.013	0.313	0.998	0.018	0.045	0.997
**CBZ**	0.003	0.008	0.998	0.017	0.050	0.995
3-OH CBZ	0.010	0.052	0.998	0.027	0.068	0.995
10-OH CBZ	0.004	0.012	0.997	0.018	0.053	0.995
EP-CBZ	0.003	0.010	0.995	0.020	0.059	0.995
**DIC**	0.009	0.086	0.999	0.016	0.040	0.996
4-OH DIC	0.002	0.007	0.997	0.039	0.781	0.994
**IBU**	0.833	2.25	0.997	0.833	2.25	0.996
1-OH IBU	0.164	0.820	0.998	0.179	0.446	0.996
2-OH IBU	0.003	0.009	0.998	0.023	2.326	0.998
CBX-IBU	1.020	2.25	0.999	0.018	1.761	0.998
**SMX**	0.008	0.024	0.997	0.417	1.667	0.996
AcSMX	0.007	0.070	0.999	0.049	0.196	0.996

Parent compounds are marked in bold.

**Table 2:** Recovery (%) and precision, expressed as relative standard deviation (RSD%), for lettuce and carrot matrices at three spiking levels.

Compound	Lettuce						Carrot					
	2.5 (ng g^−1^ dw)		40 (ng g^−1^ dw)		100 (ng g^−1^ dw)		2.5 (ng g^−1^ dw)		40 (ng g^−1^ dw)		100 (ng g^−1^ dw)	
	R (%)	RSD (%)	R (%)	RSD (%)	R (%)	RSD (%)	R (%)	RSD (%)	R (%)	RSD (%)	R (%)	RSD (%)
**CAF**	94	12	34	0	37	11	115	10	41	4	33	7
PX	80	21	49	5	28	6	55	15	41	6	38	9
**CBZ**	66	22	47	9	55	7	20	24	19	1	27	8
3-OH CBZ	48	16	48	7	51	8	37	3	52	10	47	14
10-OH CBZ	43	18	45	8	51	10	19	25	17	4	26	8
EP-CBZ	48	18	20	1	21	10	17	18	26	24	19	3
**DIC**	58	25	24	16	27	6	62	23	24	14	21	3
4-OH DIC	67	23	61	2	38	4	64	12	34	18	31	7
**IBU**	13	27	23	1	31	7	20	30	48	21	60	28
1-OH IBU	61	28	45	9	53	9	56	7	48	9	40	11
2-OH IBU	55	14	44	5	41	7	43	18	42	11	37	9
CBX-IBU	98	16	43	5	52	9	142	19	48	16	42	13
**SMX**	21	30	21	3	25	8	16	22	26	17	27	2
AcSMX	71	30	51	6	45	9	51	11	44	14	42	13

Parent compounds are marked in bold.

**Table 3:** Accuracy (%) and matrix effect (%) for lettuce and carrot matrices at three spiking levels.

Compound	Lettuce						Carrot					
	2.5 (ng g^−1^ dw)		40 (ng g^−1^ dw)		100 (ng g^−1^ dw)		2.5 (ng g^−1^ dw)		40 (ng g^−1^ dw)		100 (ng g^−1^ dw)	
	A (%)	ME (%)	A (%)	ME (%)	A (%)	ME (%)	A (%)	ME (%)	A (%)	ME (%)	A (%)	ME (%)
**CAF**	87	−27.6	95	−34.8	102	−0.34	81	−62.4	99	−37.6	107	−35.0
PX	88	−13.2	94	−19.9	103	−0.22	84	−54.2	95	−64.3	99	−27.1
**CBZ**	86	−7.07	92	−1.77	101	−0.01	82	−28.8	96	−0.81	98	−6.33
3-OH CBZ	89	−15.9	93	−11.9	100	−0.10	83	−44.8	98	−24.4	100	−10.6
10-OH CBZ	81	−7.41	96	−5.00	99	−0.03	85	−2.73	97	−7.37	102	−2.93
EP-CBZ	84	−30.8	91	−12.1	97	−0.10	87	−11.2	100	−19.8	104	−8.05
**DIC**	82	−15.3	91	−39.0	98	−0.31	88	−53.5	92	−55.2	107	−15.8
4-OH DIC	83	−32.0	94	−11.7	100	−0.10	86	−6.27	94	−26.02	105	−4.11
**IBU**	88	−11.2	93	−8.76	101	−0.32	89	−35.7	93	−22.2	103	−8.41
1-OH IBU	87	−9.76	95	−4.92	104	−0.02	91	−28.7	91	−0.08	106	−0.42
2-OH IBU	87	−6.06	98	−4.31	106	−0.04	92	−45.6	100	−27.7	101	−6.85
CBX-IBU	85	−7.98	96	−0.45	101	−0.01	90	−49.5	97	−6.49	97	−1.24
**SMX**	84	−20.3	94	−20.8	99	−0.19	85	−79.3	98	−75.7	99	−26.1
AcSMX	81	−1.81	97	0.02	98	−0.01	82	−33.4	99	−29.9	101	−1.32

Parent compounds are marked in bold.

**Table 4:** Comparison with other methods reported in the literature.

Compounds	Sample Amount (g)	Extraction Technique	Time (min)	Solvent (mL)	Clean-Up Step	Analytical Determination	MQL (ng g^−1^ dw)	Reference
1 CAF, 1 analgesic, 8 antibiotics, and 1 anticonvulsant	0.5	QuEChERS	3.5	ACN, MeOH 5	-	LC-MS/MS	0.7–8.0 (MDL)	[20]
1 CAF, 1 analgesic, 8 antibiotics, and 1 anticonvulsant	0.5	PLE	20	ACN, MeOH 40	SPE	LC-MS/MS	1.9–15.8 (MDL)	[20]
7 antibiotics	0.25–0.5	UAE	6	18 (Alkaline solution with Mg^2+^)	SPE	LC-MS/MS	2–10	[17]
1 anticonvulsant	0.5	PLE	27	n.d. (Acetone, hexane)	-	GC-MS	7.6–61.7	[19]
11 antibiotics	1.0	UAE	30	20 (ACN)	SPE	LC-MS/MS	0.2–6.25	[18]
10 antibiotics and 6 of their metabolites	1.0	UAE	30	20 (MeOH)	SPE	LC-MS/MS	0.2–9.2	[24]
26 antibiotics	10	QuEChERS	27	10 (ACN, MeOH)	-	LC-MS/MS	0.02–1.5	[23]
20 antibiotics	10	QuEChERS	35	10 (ACN, MeOH)	-	LC-MS/MS	0.33–2.92 (MDL)	[22]
1 CAF, 2 NSAIDs, 1 antibiotic, 1 anticonvulsant, and 9 of their main metabolites	0.5	UAE	27	12 (Acetone)	d-SPE	LC-MS/MS	0.007–2.25	Proposed methodology

n.d.: no data.

## Data Availability

Data will be made available on request.

## Supplementary Materials

The following supporting information can be downloaded at: https://www.mdpi.com/article/10.3390/molecules29153471/s1, Figure S1: Chromatogram obtained from a spiked lettuce (10 ng g^−1^ dw); Table S1: Box–Behnken design matrix for the optimisation of clean-up sorbent amount; Table S2: Box–Behnken design matrix for the optimisation of extraction solvent volume, UAE time extraction, and number of extraction cycles; Table S3: Analytes detected in leafy and root vegetables from local markets; Table S4: Physical–chemical properties of the target compounds; Table S5: LC-MS/MS conditions and retention times for the target compounds.
