# Supplementary material for: Multiclass Analysis for the Determination of Pharmaceuticals and Their Main Metabolites in Leafy and Root Vegetables
Source: Molecules. 2024 Jul 24;29(15):3471. doi: 10.3390/molecules29153471 (PMC11313980; doi:10.3390/molecules29153471)
Supplement: Supplementary file 1 [file molecules-29-03471-s001.zip › molecules-3120802-supplementary.pdf]

Multiclass Analysis for the Determination of Pharmaceuticals and Their Main Metabolites in Leafy and Root Vegetables

Carmen Mejías, Marina Arenas, Julia Martín, Juan Luis Santos, Irene Aparicio, Esteban Alonso

Corresponding author: Julia Martín Bueno

Address: Departamento de Química Analítica, Escuela Politécnica Superior, Universidad de Sevilla. C/ Virgen de África, 7, E–41011 Seville, Spain

E-mail: jbueno@us.es

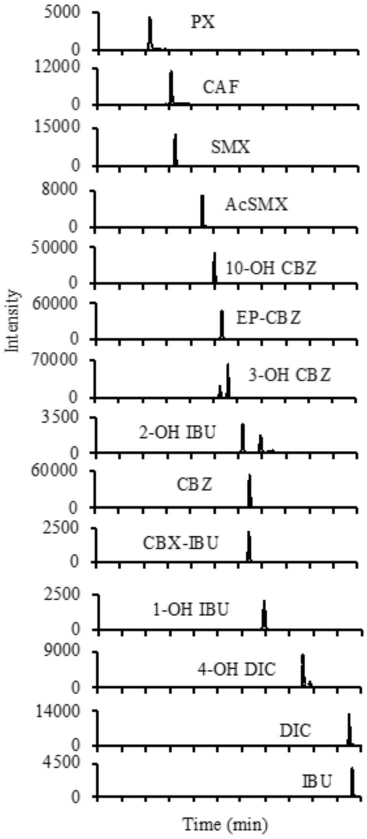

Figure S1. Chromatogram obtained from a spiked lettuce (10 ng g^{-1} dw).

Table S1. Box–Behnken design matrix for the optimization of clean-up sorbent amount.

Experiment	C18 amount (g)	PSA amount (g)	Florisil amount (g)
1	0.4	0.4	0.4
2	0.4	0.4	0.4
3	0	0.4	0
4	0.8	0	0.4
5	0.4	0	0.8
6	0.8	0.8	0.4
7	0.8	0.4	0.8
8	0.4	0.8	0
9	0.4	0	0
10	0.4	0.8	0.8
11	0	0	0.4
12	0.4	0.4	0.4
13	0	0.4	0.8
14	0.8	0.4	0
15	0	0.8	0.4

Table S2. Box–Behnken design matrix for the optimization of extraction solvent volume, UAE time extraction and number of extraction cycles.

Experiment	Acetone (1% v/v, formic acid) volume (mL)	Time of extraction (min)	Number of extractions
1	6	10	1
2	6	15	2
3	4.5	10	2
4	4.5	5	1
5	3	10	1
6	6	5	2
7	6	10	3
8	4.5	5	3
9	3	10	3
10	4.5	15	1
11	4.5	10	2
12	4.5	10	2
13	4.5	15	3
14	3	15	2
15	3	5	2

Table S3. Analytes detected in leafy and root vegetables from local markets.

Compound	Carrot (ng g^{-1} dw)			Lettuce (ng g^{-1} dw)		
	S1	S2	S3	S1	S2	S3
**CAF**	4.088	<MQL	<MQL	-	-	8.960
PX	-	<MQL	-	-	-	-
**CBZ**	<MQL	0.143	-	-	-	-
3-OH CBZ	<MQL	-	-	0.110	-	-
10-OH CBZ	<MQL	0.094	0.112	-	-	-
EP-CBZ	0.282	-	-	-	4.472	-
**DIC**	<MQL	-	-	-	-	-
4-OH DIC	-	-	-	-	-	-
**IBU**	4.020	-	-	-	-	-
1-OH IBU	<MQL	-	-	-	-	-
2-OH IBU	-	-	-	-	-	-
CBX- IBU	-	-	-	-	-	-
**SMX**	7.818	1.798	5.792	-	0.590	-
AcSMX	0.556	4.466	1.044	-	-	-

Parent compounds are marked in bold; -: not detected.

Table S4. Physical-chemical properties of the target compounds.

Group	Compound	Molecular weight (g mol^{-1})	pK$_a$	Log K$_{ow}$	Structure
Caffeine	**1,3,7-trimethylxanthine (caffeine) (CAF)**	194.19	10.4	-0.1	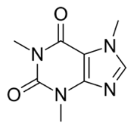
	1,7-dimethylxanthine (PX)	180.16	8.5	-0.2	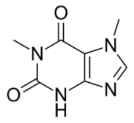
Antiepileptic drug	**Carbamazepine (CBZ)**	236.27	13.9	2.5	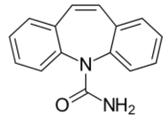
	3-Hydroxycarbamazepine (3-OH-CBZ)	252.27	9.19	2.41	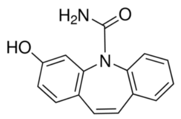
	10,11-Dihydro-10-hydroxycarbamazepine (10-OH-CBZ)	254.28	12.8	0.93	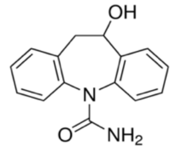
	Carbamazepine-10,11-epoxide (EP-CBZ)	252.27	16.0	1.0	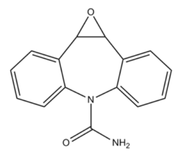
Antiinflammatory drug	**Diclofenac (DIC)**	295.15	4.2	4.5	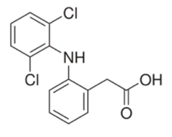
	4-Hydroxydiclofenac (4-OH-DIC)	312.15	4.17	3.97	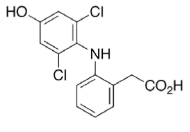
	**Ibuprofen (IBU)**	206.28	4.9	4.0	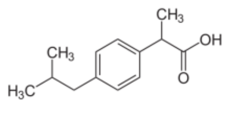
	1-Hydroxyibuprofen (1-OH IBU)	222.28	4.55	2.69	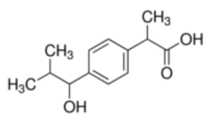
	2-Hydroxyibuprofen (2-OH IBU)	222.28	4.63	2.37	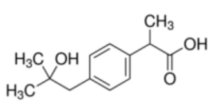

	Carboxyibuprofen (CBX-IBU)	236.26	3.97	2.78	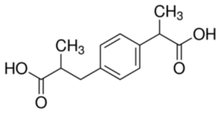
Antibiotic	**Sulfamethoxazole (SMX)**	253.28	5.7	0.9	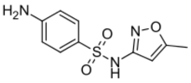
	N^4-Acetylsulfamethoxazole (AcSMX)	295.31	5.54	1.18	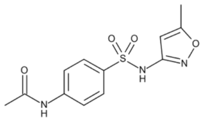

Parent compounds are marked in bold.

Table S5. LC-MS/MS conditions and retention times for the target compounds.

Compound	Precursor ion *(m/z)*	Product ions (quantifier/qualifier) *(m/z)*	CE (eV)	Ratio	Polarity	Retention time (min)
**CAF**	195.1	138.0/42.0	20/24	10.9	Positive	5.32
PX	181.1	124.0/42.1	20/0	2.0	Positive	3.81
**CBZ**	237.1	194.0/179.0	20/40	29.4	Positive	9.07
3-OH CBZ	253.1	209.9/167.0	20/48	28.9	Positive	10.20
10-OH CBZ	255.3	194.1/237.1	20/4	98.7	Positive	9.07
EP-CBZ	253.1	180.0/235.9	36/8	56.3	Positive	9.51
**DIC**	296.0	213.9/249.8	40/12	49.1	Positive	20.10
4-OH DIC	312.0	229.9/266.0	40/8	49.3	Positive	16.34
**IBU**	205.1	205.1/161.1	0/4	8.8	Negative	20.33
1-OH IBU	240.2	205.0/163.0	8/20	64.4	Positive	11.60
2-OH IBU	240.2	205.0/107.0	12/36	24.1	Positive	13.12
CBX- IBU	254.1	218.9/117.1	8/48	56.0	Positive	12.08
**SMX**	254.1	92.1/65.0	32/28	8.0	Positive	5.86
AcSMX	296.1	134.0/197.9	28/20	63.8	Positive	8.06
CAF- $^{13}C_3$	198.2	140.0/112.0	20/24	22.9	Positive	5.32
IBU- d_3	227.2	158.9/91.0	4/12	18.8	Negative	20.33

Parent compounds are marked in bold; CE: collision energy.
